# PCR-DGGE analysis of fungal community in manufacturing process of a traditional Iranian cheese

**Published:** 2018-06

**Authors:** Mohaddeseh Ramezani, Seyed Masoud Hosseini, Seyed Abolhassan Shahzadeh Fazeli, Mohammad Ali Amozegar, Javad Fakhari

**Affiliations:** 1 Department of Microbiology and Microbial Biotechnology, Faculty of Life Sciences and Biotechnology, Shahid Beheshti University, Tehran, Iran; 2 Microorganisms Bank, Iranian Biological Resource Centre (IBRC), ACECR, Tehran, Iran; 3 Extremophiles Laboratory, Department of Microbiology, Faculty of Biology and Center of Excellence in Phylogeny of Living Organisms, College of Sciences, University of Tehran, Tehran, Iran; 4 Department of Molecular and Cellular Biology, Faculty of Basic Sciences and Advanced Technologies in Biology, University of Sciences and Culture, Tehran, Iran

**Keywords:** Traditional cheese, DGGE, Liqvan cheese, Fungal diversity

## Abstract

**Background and Objectives:**

The microbial communities of traditional milk-based food are of great importance in its manufacturing process, especially when using raw milk with natural cultures. Liqvan (Lighvan or Levan) is a traditional Iranian buried cheese, which is made from raw ewe’s milk without a starter addition. The aim of this study was to explore the fungal active population during this cheese manufacturing process by comparing DNA and RNA based culture independent method Denaturing Gradient Gel Electrophoresis (DGGE).

**Materials and Methods:**

Four samples of each milk, curd and ripened cheese were collected from Liqvan village located in East Azerbaijan province of Iran. Total DNA and RNA of each sample were extracted and PCR amplicons of D1 region of 26S rRNA gene was targeted for DGGE analysis. This method applied at both DNA and RNA levels in order to examine which taxonomic groups of fungi are active at each step of ripening.

**Results:**

DGGE profiles of yeast amplicons showed different results between extracted DNA and RNA during ripening process. However, the main group that is present in all stages of ripening process belongs to the genus *Candida* although *Kluyveromyces, Pichia, Galactomyces, Saccharomyces* and *Cryptococcus* are most abundant fungi.

**Conclusion:**

As no starter culture added to Liqvan cheese it seems fungal diversity are mainly rely on the indigenous microbiota of milk. Furthermore, the percentage of the dominant fungal genera from the total sequences differed among DNA and cDNA libraries.

## INTRODUCTION

Dairy products fulfill many of current dietary needs and their consumption has been recently increased. Different tastes and textures of these products are mainly related to their complex microbial populations. Meanwhile, cheeses as a milk derived foods have attracted great attentions for its portability, long life, and high content of fat, protein, calcium, and phosphorus (1). Technological parameters, grazing pastures and herd type largely affect the quality of cheeses. These technical factors are more significant in cheese made from raw milk without deliberate addition of selected starter cultures. Understanding the detailed microbial composition of these kinds of cheese which are naturally present in raw milk have entice great concern lately. However, traditional microbiological methods based on culture-dependent techniques, which relates to growth ability of microorganisms in different culture media at laboratory conditions have shown bias results (2). In addition to the conventional culture based methods, various molecular DNA or RNA-based approaches are now available to survey the diversity and evolution of microbial population in foods. These are considered as indispensable tools for description, detection, identification and characterization of microorganisms in food because they are faster, more reliable and somehow cheaper than conventional culturing method that fail to reproduce ecological niches and symbiotic relationships (3). Theses culture-independent PCR and/or RT-based methods mostly includes Denaturing Gradient Gel Electrophoresis (DGGE), Single-Strand Conformation Polymorphism (SSCP), Temperature Gradient Gel Electrophoresis (TGGE), Terminal Restriction Fragment Length Polymorphism (T-RFLP), Automated Ribosomal Intergenic Spacer Analysis (ARISA) and Length Heterogeneity PCR (LH-PCR) (4, 5) that able to reveal dominant species which present in food products.

**Fig. 1.**
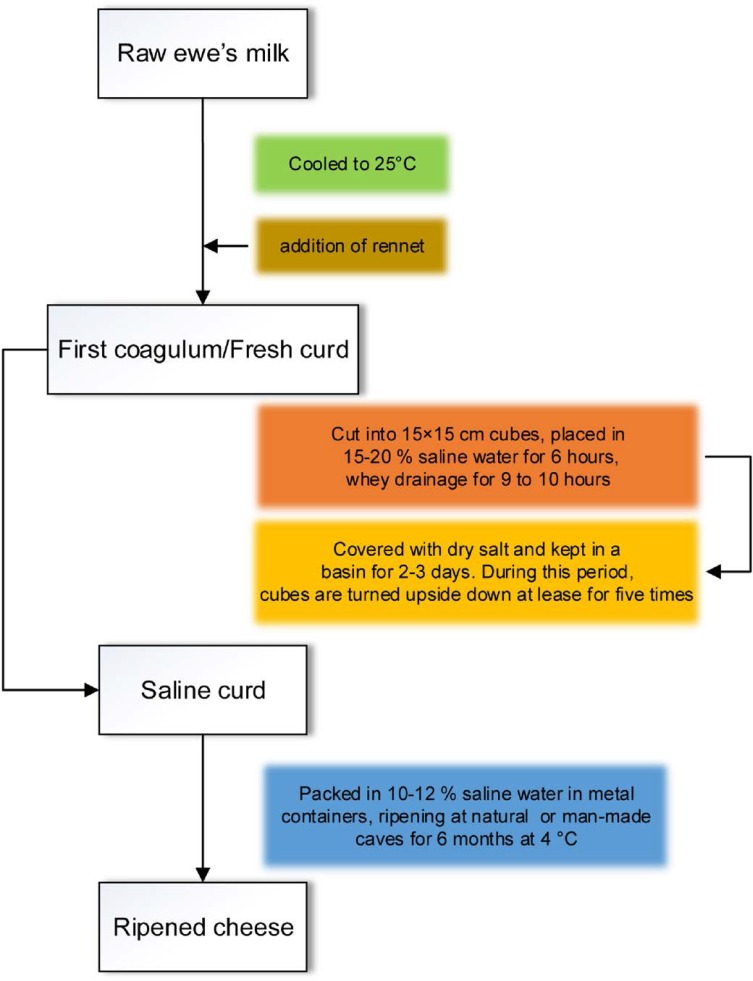
Flowchart of manufacturing procedure of traditional Iranian cheese, Liqvan

In several country sides and nomads of Iran, varieties of raw milk cheeses have been produced since long time ago. These kinds of cheeses often has stronger flavor than pasteurized milk and seem to be more natural and tasty. Among them, Liqvan (Lighvan or Levan) is the most famous, semi-hard, feta like cheese, which made from raw ewe’s milk in the Liqvan village at the mountainous area of East Azerbaijan province northeast of Iran. The complete process of Liqvan production steps is diagramed in Fig. 1. The production process occurs within 2 h from milking and ripening takes place in deep natural caves or man-made large holes for 6 months.

This traditional manufacturing process mainly relys on the indigenous microbiota of raw milk that maintained throughout centuries in that area. Previous studies on Liqvan diversity based on culture-dependent and DNA base DGGE methods demonstrated some dominant microbial populations in this type of cheese (6–9). Nevertheless, there is no report on its RNA based fingerprinting analysis yet.

This study compared the fungal profile of Liqvan cheese through reverse transcriptase PCR (RT-PCR)-denaturing gradient gel electrophoresis (DGGE) and DNA based denaturing gradient gel electrophoresis (DGGE) during its manufacturing process in order to survey which groups are the most dominant active populations in each step.

## MATERIALS AND METHODS

### Sampling collection and conditions.

Four different samples of milk, curd and cheese (A to D) were picked up at traditional farmhouses in Liqvan village. Milk and curds are directly transferred to the laboratory but cheese samples collected from the same batch after 180 days of preparation and ripening. The samples Transported in refrigerator and stored at 4°C.

### DNA/RNA extraction of fungal population.

Two milliliters of milk samples were centrifuge for 10 min at 14,000 × g under refrigerated condition (4°C). The supernatant, that contains fat layer, was discarded and cell pellets were resuspended in 1ml PBS solution. The Mixture was centrifuged at 14,000 × g at 4°C for 10 min and pellets were resuspended at 100 μl lysozyme solution (20 mg/ml). The mixture incubated at 37°C for one hour. The lysate then extracted by CinnaPure ONE kit (CinnaGen, Iran) according to manufacturer’s instructions. In case of curds and cheeses, five grams of samples were homogenized with 20 ml of PBS. One ml of this solution transferred to 1.5 ml micro-tubes afterwards and same procedure of milk followed.

After this, each resulting nucleic acids divided in two tubes. For DNA purification, RNase treatment was performed with 3 microliters of RNase A solution (Thermoscientific, Lithuania) and followed by incubation overnight at 37°C. In case of RNA, the resulted nucleic acid treated with DNase I solution that provided in the CinnaPure ONE kit and incubated at 37°C for 30 min. Complete DNA digestion was confirmed using 1 μl of extracted RNA with primers NL1 and LS2 (10, 11). At this step, when a PCR product was obtained, the DNase treatment was repeated. The resulting DNA/RNA was quantified using the PikoDrop 100 spectrophotometer (Alpha Biotech UK) and standardized at 100 ng/μL.

### Preparation of cDNA library.

One-hundred nanogram of RNA for each sample was subjected to reverse transcription using Viva 2-steps RT-PCR Kit with M-MuLV RT/Taq DNA Polymerase (Vivantis, Malaysia). The total RNA was added to 1 μl of oligo d (T)_18_ (40 μM), 10 μM LS2 primer and 1 μl of 10 mM dNTPs mix and the final volume was reached to 10 μl by nuclease free water. The mixture was then incubated at 65°C for 5 minutes and chilled on ice for two minutes. The RT was carried out at 42°C for 1 h using M-MuLV Reverse Transcriptase. The PCR reaction was performed in a MyCycler (BioRad, Hercules, USA) from three biological replicates of each batch as a template.

### PCR amplification and DGGE analysis.

Fungi D1 region of 26S rRNA gene amplification was carried out with primers NL1-GC(5′-GCCATATCAATAAGCGGAGGAAAAG-3′) and LS2 (5′- ATTCCCAAACAACTCGACTC-3′) (10). The amplification cycles and reaction mixtures were as follows: for each sample, a 25 μl PCR mixture was prepared containing 100 ng DNA or cDNA, 1X buffer PCR, 2 mM MgCl_2_, 0.2 mM of each deoxynucleoside tri-phosphates, 2 U of Taq polymerase, and 0.2 μM of each primer. The cycle was carried out at 95°C for 5 min, 35 cycles of denaturation at 95°C for 1 min, annealing at 42°C for 1 min and extension at 72°C for 1 min and a single final extension at 72°C for 10 min.

The PCR products of DNA/cDNA populations were analyzed by denaturing gradient gel electrophoresis (DGGE) technique using DCode system apparatus (BioRad, Hercules, CA, USA). Polyacrlamide gels (8% W/V, Acrylamide-Bisacrylamide 37.5:1, 0.8 mm thickness) were prepared using 40–60% formamide denaturing gradients and the electrophoresis proceeded at 120 V and 60°C for 4.5h.

Identification of fungal population was carried out by cutting the DNA bands on DGGE gels. The DNA then eluted from gels with 50μl water and stored overnight at 4°C. These extracted DNA was amplified again with non GC-clamp primers and sequenced by Bioneer, Korea.

## RESULTS

As many as 11 different bands corresponding to fungal DGGE analysis are summarized in Table 1. Yeast and molds population during Liqvan cheese manufacturing were analyzed by using D1 region of 26S rRNA gene, that amplified from isolated DNA and retro-transcribed RNA (cDNA) from milk, saline curd and ripened 180-days cheese. All the bands were checked by reamplification and sequencing. Identification of the bands excised from DGGE gels were achieved based on blast comparison in GenBank.

**Table 1.** Band sequencing results of fungi DGGE analysis

**Row**	**Closest relative**	**% identity**	**Accession number**
1	*Candida pararugosa*	99%	KF912816
2	*Candida sake*	99%	KR055663
3	*Candida zeylanoides*	100%	KC442252
4	*Cladosporium ramotenellum*	98%	KM589488
5	*Cryptococcus magnus*	100%	KF891470
6	*Debaryomyces hansenii*	98%	NG_042634
7	*Galactomyces candidum*	99%	KM115152
8	*Kluyveromyces lactis*	99%	KJ183045
9	*Kluyveromyces marxianus*	97%	KC512907
10	*Pichia fermentans*	99%	KM589456
11	*Saccharomyces cerevisiae*	97%	HM191660

### DGGE analysis of milk samples.

**Fig. 2.**
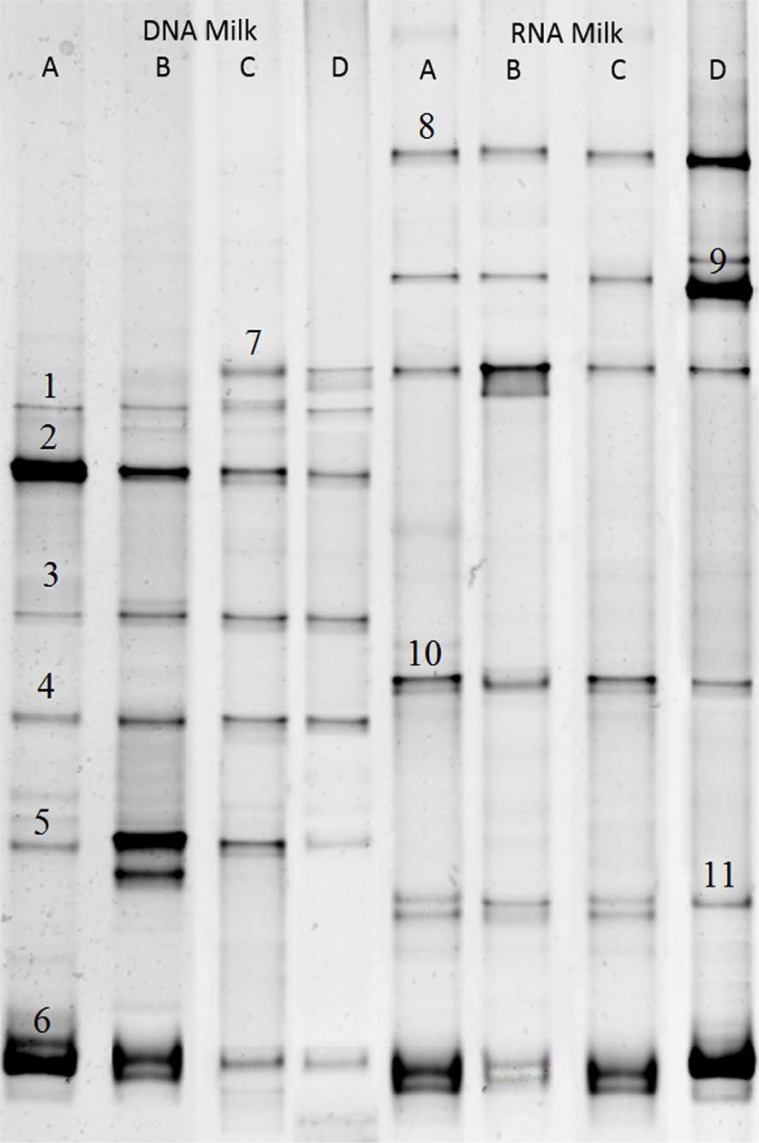
DGGE 26S rDNA and RNA profile of fungal populations in milk samples Bands were identified as Number: 1; *Candida sake*, 2; *Debaryomyces hansenii*, 3; *Kluyveromyces lactis*, 4; *Saccharomyces cerevisiae*, 5; *Kluyveromyces marxianus*, 6; *Pichia fermentans*, 7; *Candida zeylanoides*, 8; *Candida pararugosa*, 9; *Galactomyces candidum*, 10; *Cryptococcus magnus* and 11; *Cladosporium ramotenellum*.

DGGE bands from fungal identification of milk samples at DNA/cDNA levels demonstrate in Fig. 2. In milk DNA samples, bands related to *Debaryomyces hansenii* (Band No. 2), *Kluyveromyces lactis* (Band No. 3), *Candida sake* (Band No. 1), *Saccharomyces cerevisiae* (Band No. 4), *Kluyveromyces marxianus* (Band No. 5) and *Pichia fermentans* (Band No. 6) are presented in all samples. While band number 7 was only traced in samples C and D.

Whereas DGGE pattern at RNA level was different and *P. fermentans* (Band No. 6), *Candida zeylanoides* (Band No. 7), *Candida pararogosa* (Band No. 8), *Galactomyces candidum* (Band No. 9) and *Cryptococcus magnus* (Band No. 10), and *Cladosporium ramotenellum* (Band No. 11) are predominant active organisms.

The comparison between these results display that notwithstanding some fungal DNA may more concentrate at milk samples, the active population in first step of ripening is not necessarily associated with this.

### DGGE analysis of curd samples.

**Fig. 3.**
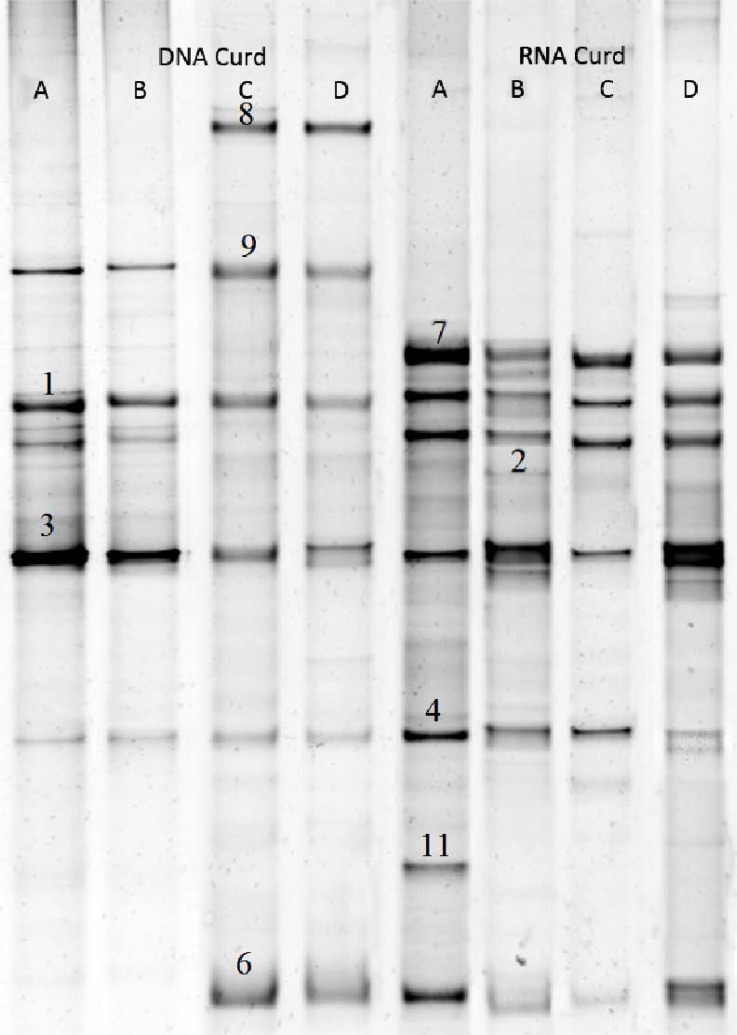
DGGE 26S rDNA and RNA profile of fungal populations in curd samples Bands were identified as Number: 1; *Candida sake*, 2; *Debaryomyces hansenii*, 3; *Kluyveromyces lactis*, 4; *Saccharomyces cerevisiae*, 5; *Kluyveromyces marxianus*, 6; *Pichia fermentans*, 7; *Candida zeylanoides*, 8; *Candida pararugosa*, 9; *Galactomyces candidum*, 10; *Cryptococcus magnus* and 11; *Cladosporium ramotenellum*.

The DGGE results corresponding to curd samples are presented in Fig. 3. It is important to mention that *C. sake* (Band No. 1), *K. lactis* (Band No. 3) and *G. candidum* (Band No. 9) are predominant population in all samples at DNA. *S. cerevisiae* (Band No. 4) also present with less intensity. Besides, *P. fermentans* (Band No. 6) and *C. pararogosa* (Band No. 8) attended in samples C and D. Howbeit this template is not repeated at RNA level and bands correspond to *C. sake* (No. 1), *D. hansenii* (No. 2), *K. lactis* (No. 3), *S. cerevisiae* (No. 4), *P. fermentans* (No. 6) and *C. zeylanoides* (No. 7) are active populations. Of course, there are differences between samples, so that in curd sample A, *C. ramotenellum* is a part of alive population in the process.

Consequently, it seems that there is a relation between presence and activity of *C. Sake*, *K. lactis* and somehow *S. cerevisiae* at this step yet it does not meet about other fungal species.

### DGGE analysis of cheese samples.

**Fig. 4.**
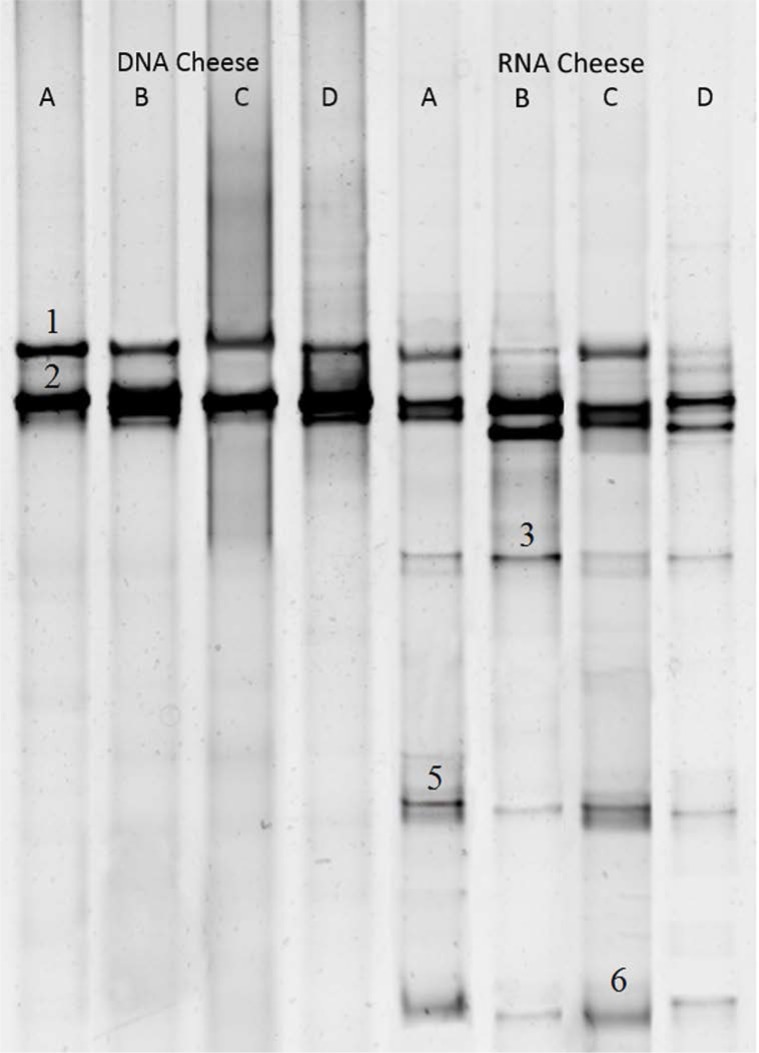
DGGE 26S rDNA and RNA profile of fungal populations in cheese samples Bands were identified as Number: 1; *Candida sake*, 2; *Debaryomyces hansenii*, 3; *Kluyveromyces lactis*, 4; *Saccharomyces cerevisiae*, 5; *Kluyveromyces marxianus*, 6; *Pichia fermentans*, 7; *Candida zeylanoides*, 8; *Candida pararugosa*, 9; *Galactomyces candidum*, 10; *Cryptococcus magnus* and 11; *Cladosporium ramotenellum.*

Cheese look to follow same pattern between extracted DNA and RNA and bands correlate to *C. Sake* and *D. hansenii* (Band numbers 1 and 2, respectively) are the predominant ones in all samples (A to D). Albeit results showed more divergence among RNA samples as bands related to *K. lactis* (No. 3), *K. marxianus* (No. 5) and *P. fermentans* (No. 6) are further detected in them. Fig. 4. illustrates the DGGE results of cheese.

## DISCUSSION

Molecular fingerprinting methods have been described as useful techniques for monitoring dominant microbial diversity and dynamics in dairy environments (12). Among these techniques, DGGE has been successfully developed and used in milk-based products especially those that based on raw milk microbiota (3, 10, 11).

Liqvan is a traditional Iranian cheese that originating from a specific area of North-East part of Iran and its quality and special characteristics are exclusively due to its production way in a particular geographic region. As this cheese made from raw milk without starter cultures, it seems that ripening and typical sensorial properties of it is mainly rely on its indigenous microbiota. On the other hand, Liqvan traditional manufacturing way of production maintained for centuries so identification and characterization of its indegenus microorganisms is an important issue. Besides, the first step toward protecting microbial diversity in traditional regional food is elucidate active microfloral evolution during manufacturing and ripening processes especially for safety and quality of final products (13).

Most of previous studies on Liqvan cheese had focused on bacterial community monitoring (6–9), however there are some studies which included fungi population too. Nevertheless, surveying diversity and activity of yeast and molds population has not been worked before. Moreover, no RNA-DGGE based analysis on this type of cheese has been reported yet.

D1 region of fungal 26S rRNA gene is one of the best choices for identification of fungal communities at DGGE based analysis, as it has suitable length and species-specific heterogeneity. In this study, both DNA- and RNA-based DGGE relying D1 segment from 26S rRNA gene used in order to indicate dominant fungal population during ripening process of Liqvan cheese. This could lead to make a proper comparison between active and inactive fungal microbiota during manufacturing procedure.

The main fungi that were detected in all samples by DGGE included three different species of *Candida (C. pararugosa, C. sake,* and *C. zeylanoides)*, two species of *Kluyveromyces (K. marxianus, K. lactis)*, *C. ramotenellum, C. magnus, D. hansenii, G. candidum, P. fermentans* and *S. cerevisiae. Candida* spp. representing the main numerically fungal group, which found in all samples that estimated 27.3% of total isolates, followed by *Kluyveromyces* that contributed 18.2% of total population. However, the percentage of the dominant fungal society among DNA and cDNA libraries are different. For instance, genera *C. sake, D. hansenii, K. lactis, S. cerevisiae* and *K. marxianus* were present in a range of 100% of 26S rDNA library in milk while they have no activity in RNA-DGGE samples and instead other genera include *C. zeylanoides, C. pararogosa, G. candidum, C. magnus* and *C. ramotenellum* constituted the active populations in milk. The only population which present in both milk DNA and cDNA was *P. fermentans*. Additionally, in curd DNA samples, *C. pararugosa* and *G. candidum* are present with 100% and 50% incidence; respectively, while they have no activity during curd ripening. Interestingly, relatively high ratio of reads at both DNA and RNA levels in cheese were assigned to *C. sake* and *D. hansenii*.

In conclusion, since no starter culture added to Liqvan cheese, it is known that the heterogeneity of fungi identified in current analysis, are mainly rely on indigenous microbiota of milk. It seems that despite the differences between extracted DNA during ripening process, at RNA level *P. fermentans, C. zeylanoides, C. pararogosa, G. candidum, C. magnus* and *C. ramotenellum* are active fungal organisms that are mainly involved in converting process of milk to curd. Furthermore, *C. sake, D. hansenii, K. lactis, S. cerevisiae, P. fermentans* and *C. zeylanoides* are the active groups that cooperates in modification of curd to cheese. Regarding cheese samples, it is appear that due to the end of cheese fermentation process and ripening, the native milk fungal community have a tendency to decrease in number and diversity. Notwithstanding the fact that these all processes are not occurred only by fungi and managed with a close relationship with an active bacterial population.
